# Effects of Training and Detraining on Physical Fitness, Physical Activity Patterns, Cardiovascular Variables, and HRQoL after 3 Health-Promotion Interventions in Institutionalized Elders

**DOI:** 10.1155/2010/486097

**Published:** 2011-02-15

**Authors:** Alexandrina Lobo, Joana Carvalho, Paula Santos

**Affiliations:** Research Center in Physical Activity, Health and Leisure, Faculty of Sport, University of Porto, Rua Dr. Plácido Costa 91, 4200-450 Porto, Portugal

## Abstract

The purpose of this study is to assess the effects of different strategies of health on the levels of physical activity (PA), physical fitness (PF), cardiovascular disease (CVD) risk factors and quality of life (QoL) of the institutionalized elderly. Concurrently studies were made of the effect of detraining on these same variables. In this investigation we carried out a prospective longitudinal study with an experimental design, with 1 year plus 3 months of a detraining period. *Methodology*. (a) A questionnaire with socio-demographic characteristics and a QoL scale (MOS SF-36); (b) Functional Fitness Test to assess PF; (c) An MTI Actigraph to evaluate the PA; (d) Biochemical analysis of blood, blood pressure and bio-impedance. *The Main Results Indicated That*: (i) ST significantly improved strength and body flexibility and AT the aerobic endurance, agility/dynamic balance and lower strength and flexibility; (ii) Implications of detraining were more evident on the PA groups in the lower body flexibility, which is associated with agility/dynamic balance and lower strength in the AT group; (iii) Cardiovascular variables improved significantly especially blood pressure, cholesterol and glucose in the ST and HDL in the AT group; not having undergone significant changes with the detraining. The results of this thesis contribute positively to highlight the importance of PA in the promotion of health, prevention and reduction of CVD risk factors and the improvement of the PF and QoL.

## 1. Introduction

In developed countries, people aging 65 and older constitute approximately 20% of the population and are the fastest-growing segment of the population [1]. With this growing aging population, the number of institutionalization will likely increase in coming years. Being relocated to an assisted living facility can result in functional and health disturbances and worsen quality of life in elders. This may be attributed to or worsened by lack of regular physical activity. Appropriate exercise programs may be an important component of quality of life in this group of transitional frail elders [2, 3].

Previous studies suggest that participation in a regular exercise program can be an effective intervention to reduce/prevent a number of functional declines associated with aging [4, 5]. In this regard, current guidelines point out the importance of aerobic exercise in the maintenance and improvement of various aspects of cardiovascular function and health and strength training in the attenuation of muscle strength declines associated with normal aging [6, 7]. 

Moreover, considering that cardiovascular diseases (CVD) are the major cause of death in the elders, sustaining older adults' ability to live independently as well as reducing blood pressure (BP), plasma lipoprotein lipid profiles, and body weight via healthy lifestyles interventions are very important goals of public health, geriatrics, and gerontology. Although there is sufficient data showing that regular physical activity (PA) is associated with improvements in cardiovascular health among older adults [8–10], additional information is necessary concerning the effectiveness of different types of heath promotion and exercise interventions.

Furthermore, regarding that a number of studies have indicated that both morphologic and functional adaptations can decrease even after short detraining periods [11], it seems of interest to know whether the heath promotion interventions benefits are maintained when detraining occurs, due to unexpected causes such as, illness, vacations, or others. In fact, despite evidence of physiological decline during detraining, there is not enough data suggesting how long the beneficial effects of training are maintained, how this affects the health related quality of life (HRQoL) and how functional fitness changes following the cessation of exercise intervention in institutionalized elders [12].

Most studies of detraining in elders have examined the effects after exercise intervention and focused on different performance measures of physical function, resembling the effects on functional fitness response [13–15]. While some studies have shown similar decreases in strength, aerobic endurance, and agility/dynamic balance [11, 16], others have showed that increases in muscle strength after training can be maintained after 24 weeks [11], 27 weeks [17], and 31 weeks [18] of detraining in older adults. According to the literature the initial levels of physical activity and functional capacity, gender differences, duration of the detraining period, age, and the evaluation method used could be possible explanations for the discrepancy between studies. Few studies have reported detraining evidence after different types of interventions [12, 19] and those have not addressed HRQoL and cardiovascular health variables. 

Therefore, the purposes of this study were to investigate the effects of 1 year of 3 different health intervention programs (aerobic training, strength training, and health education) on physical fitness, PA patterns, cardiovascular health variables, and HRQoL. In addition, the effect of 3 months of detraining was also analysed.

## 2. Methods

### 2.1. Subjects

Subjects were recruited from 8 long-term care institutions (nursing homes) randomly selected in the Oporto area (North of Portugal). Two hundred nineteen elderly subjects aging 66 to 92 volunteered to participate in this study. At the screening, participants completed a health history questionnaire to record past and present conditions and medication, as well as a sociodemographics and years of institutionalization questionnaire. Participants were considered eligible for inclusion if they had scored 24 or more on the mini-mental state examination (without mental dysfunction) [20] with a Barthel index 40 or more (without several dependence) [21] and also if they were not engaged in regular exercise of moderate to vigorous intensities for 20 minutes or more at least twice a week in the past 3 years [22]. Participants were excluded if they had any medical or physical limitations for testing or training and were without medical assistance or control drugs. There were 11 elders with osteoporosis, 24 with diabetes, 7 with respiratory illness, 19 with hypertension, 44 with hypercholesterolemia, and 5 with arthritis. In general, each subject received 6 different drugs (range 4–11) daily, including supplements such as calcium, vitamin B, diuretics, laxatives, heart drugs, antihypertensive, antidepressants, and painkillers.

Before conducting the study, all participants received a complete explanation of the purpose, risks, and procedures of the investigation and gave their written consent. The investigation was in full compliance with the Helsinki declaration of 1975, as revised in 2004. All methods and procedures were approved by the Institutional Review Board. The elderly subjects provided written informed consent before the study.

### 2.2. Design

Due to recruitment implications according to infrastructures available on each institution, this study was not a true randomized controlled trial. Therefore, the 185 eligible subjects, from institutions registered in this study, were allocated into four groups: aerobic exercise program (AT; *N* = 49), strength training program (ST; *N* = 37), health education program (HE; *N* = 52), and control group (CG; *N* = 47). However, after randomization, 19 participants of the ST group volunteered for the control group, potentially reducing the sample size of ST program (*N* = 18). 

Data were collected by trained investigators, using always the same protocol, for all variables at baseline (M1—January 2006), after the 1-year control period (M2—February of 2007), and, finally, after 3 months of detraining (M3—May of 2007).

## 3. Health-Promotion Interventions

### 3.1. Exercise Interventions

The 1-year exercise interventions were held twice a week, each session lasted 30–40 minutes and were supervised by a qualified and trained instructor.

### 3.2. Aerobic Training

Briefly, AT sessions consisted of 10 minutes warm-up, followed by 20 minutes of aerobic workout using continuous walking and dancing using rhythmic and large muscle groups movements and ended with a 8–10 minutes of cool-down. In the first month, duration was increased from 20 to 30 minutes with an intensity of 2-3 of the adapted Borg Rating of Perceived Exertion scale (RPE) [23]. Subsequently, the intensity was gradually increased up to 4-5 in the adapted Borg RPE. Moreover, in order to evaluate the intensity of AT and detect workloads that rise above moderate intensity, the talk test was also used.

### 3.3. Strength Training

ST consisted of 2 sets of 6 exercises (each with 8 to 12 repetitions) specifically directed to the development of endurance, muscle mass, and strength of the quadriceps (using leg press and leg extension), hamstrings (using seated leg curl), trunk and arms (using women's double chest, lateral raise, and overhead press), and abdominal wall (using an abdominal machine). Movements were repeated approximately every 6 seconds, with 2 seconds for each lifting movement (concentric muscle action) and 4 seconds for each lowering movement (eccentric muscle action). During the first two weeks the training intensity was 45–50% of 1 RM in order to promote adaptation to exercise routine. From third week, the load was raised to 60–65% of 1 RM and maintained until the final of the program. The 1 RM tests were performed each 15 days until the first month and then each 4 weeks until the end of the program.

### 3.4. Health Education

For the elderly receiving HE, health professionals provided encouragement and reinforcement of the importance about PA without providing any exercise protocols to follow. These interventions covered broad domains of health care management, well-being and social awareness related to elderly people's health, and health care. Health care management was primarily related to nutritional advice, management of bone, and joint-related illness (e.g., joint pain, arthritis, and back pain) as well as other illnesses (e.g., diabetes, high blood pressure, etc.). An expositive and participative strategy was adopted for 1 hour per week sessions.

### 3.5. Detraining

Normal activities of daily living were maintained during detraining, but none of the subjects participated in any supervised exercise program. In order to control the absence of any exercise or other health-promotion interventions, they were contacted face to face by the health professionals of the institutions.

### 3.6. Control Group

Subjects allocated into CG (*N* = 66) were instructed not to change their daily living PA routines or dietary patterns during the course of the study and not to carry out any supervised PA and were informed that they would receive intervention after the end of the study. After the observation period, subjects randomized to the CG were invited to participate in specific exercise programs designed to seniors undergoing in the institution.

## 4. Measurements

### 4.1. Study Variables

#### 4.1.1. Anthropometrics

Body mass index (BMI) measure was used as an estimate of body composition. Body height was measured to an accuracy of 1 cm, with the subject in an upright position with a standard stadiometer. Body weight was measured to the nearest 0.1 kg, with subjects lightly dressed and in stocking feet. BMI was calculated using the standard formula: mass (kg)/height (m^2^). The percentage of body fat was evaluated with the bioelectrical impedance Bodystat it's the same that Stella [23]. This is an alternative easy and noninvasive method to specifically assess the percentage of fat through a low-intensity electric current that runs through the subject's body [24]. The average values of body fat were between 17 and 21% for men and 22 and 31% for women [25].

#### 4.1.2. Subjective Health-Related Quality of Life

Older adults' perceptions of their general health were assessed using the Medical Outcomes Study 36-Item Short-Form Health Study—MOS SF-36 adapted and validated for Portuguese population [26]. The MOS SF-36 is a generic measure of health condition including 36 items covering 8 dimensions: physical functioning (PF—10 items), body pain perception (BP—2 items), general health (GH—5 items), vitality (VT—4 items), social functioning (SF—2 items), emotional role limitations (EH—3 items), limitations due to physical health (PH), and mental health (MH—5 items) scores are summarized into two main domains (physical and mental component) and ranged from 0 (worst possible quality of life) to 100 (best possible quality of life). As suggested by Ware and Sherbourne [27], the instrument was applied by an interview and scores were calculated using the methods set out by the authors.

#### 4.1.3. Physical Fitness

Physical fitness was evaluated using the functional fitness test (FFT) [28], that consists of 6 validated items (and one alternative) designed to assess the physical mobility of older adults. 

Lower body strength was measured using the 30-second chair stand test. Participants were asked to sit on a 43-cm-high chair with arms crossed at the wrists and held against the chest. Participants completed as many “stand ups” as possible within 30 s. The score was the total number of stands executed through the full range of motion within 30 s. The reliability of this strength test is high (*r* = 0.92) [28].

Upper body strength was assessed using the arm curl test. Participants performed as many biceps curls as possible in 30 s, using a dumbbell. The score was the total number of handweight curls performed through the full range of motion in 30 s. The arm curl test has good relative reliability across trials (*r* = 0.80) [28]. 

Lower body flexibility was assessed using the chair sit-and-reach test. The score was the best distance achieved between the extended fingers and the tip of the toe, measured to the nearest 0.5 cm. The reliability of the chair sit-and-reach test is high (*r* = 0.96) [28].

Upper body flexibility was assessed using the back scratch test. The score was the shortest distance achieved between the extended middle fingers, measured to the nearest 0.5 cm. The reliability of this test is high (*r* = 0.92) [28]. 

Agility/dynamic balance was assessed using the 8-foot up-and-go test. The score was the shortest time to rise from a seated position, walk 2.44 m (8 ft), turn, and return to the seated position, measured to the nearest 1/10th s. The 8-foot up-and-go test has showed a high test-retest reliability of 0.90 [28].

Aerobic endurance was measured using the 6-minute walk test. Participants were asked to walk as fast as possible for 6 min with verbal encouragement given at 30-second intervals. The score was the total distance walked in 6 min along a 45.72-meter rectangular course, which was marked every 4.57 m. The reliability of this test is high (*r* = 0.91) [28].

All test stations were organized in a circuit and the same conditions were maintained for each test at all testing periods, after a demonstration by the tester, a practice trial of 2 repetitions, followed by 1 test trial. Full detailed information on test administration and protocols can be found in the work of Rikli and Jones [28].

#### 4.1.4. Blood Pressure

Resting blood pressure (BP) was measured with the Dinamap vital signs monitor (Critikon; GE Medical Systems, Milwaukee, WI), using the right arm. After being 15 minutes at supine rest in a quiet, temperature-controlled room, BP measurements were taken with the subjects seated in an upright position with the arm comfortably placed at heart level. Measurements of the diastolic (DBP) and systolic (SBP) BPs were taken within a 1-minute break, by the same investigator. The average of the 3 measurements for SBP and DBP was entered as data [29]. The measurements were performed between 8:00 AM and 11:00 AM, at least 30 minutes after meal. Furthermore, none of the subjects were smoker or under caffeine effect. Subjects were deemed to be hypertensive where their SBP was ≥140 mmHg and their DBP was ≥90 mmHg or they were on current antihypertensive drug treatment [30].

### 4.2. Biochemical Analyses

A venous blood sample for biochemical assays was withdrawn from the antecubital vein after an overnight fast for measurement of fasting blood glucose and lipid concentrations (high-density lipoprotein (HDL), low-density lipoprotein (LDL), triglycerides (TRIGs) and total cholesterol (TC)). Samples were stored at 80°C and analysed using the cholesterol esterase/oxidase enzymatic method, and triglyceride was analysed using the lipase/glycerol kinase/glycerol phosphate oxidase enzymatic method. HDL was analyzed using the homogeneous polyanion/cholesterol esterase/oxidase enzymatic method. Glucose was analyzed using the hexokinase method. Blood lipids and glucose were measured on an Olympus AU600 autoanalyser.

### 4.3. Daily Physical Activity

An accelerometer MTI Actigraph [31] was used before and after the intervention as an objective measure of daily PA over seven consecutive days. All participants were instructed about how to carry out the accelerometer that should be placed in a small nylon pouch and firmly adjusted at the person's waist using an elastic belt over the hip. A data sheet was given to each senior participant who was instructed to record the time when the monitor was attached in the morning and detached in the evening and every time he or she performed any restricted activities like showering and swimming. 

These data, summarized as counts per day, were considered to be complete if 70% of the day (1000 minutes) was recorded for at least 4 of the 5 week days, and 2 days of the weekend. For the present study, the time duration or sampling period was set at 1 minute and the output was expressed as counts per minute (counts·min^−1^). Then, for the statistical analysis we calculated the mean of the counts by hour. PA was also expressed as the average counts per hour (counts·h^−1^) over the 7 days.

To the best of our knowledge, there are no appropriate count cut-points that represent meaningful intensity categories (sedentary, moderate, and vigorous) in older adults, therefore, in the current study, activity intensities were based according to the research findings of Freedson et al. [32].

### 4.4. Statistical Analyses

All data are reported as means (±SD). Data were analysed with SPSS software (version 17.0). The level of significance was set at *P* < .05.

Shapiro-Wilk test was used to ascertain on all variables the normality of data distributions. The delta (Δ) was calculated via the standard formula: Δ  =  {(posttest score—pretest score)/pretest score}. Weight outcomes were assessed by three different moments: Δ_1_ change from baseline to 1 year, Δ_2_ weight change from 1 year to 15 months, and also Δ_3_ weight change from baseline to 15 months. 

Differences within the group in pre- versus postintervention and detraining values were performed by one-way analysis of variance (ANOVA). Scheffe was used for post hoc comparisons. Repeated measures and multivariate analyses of variance were used to examine differences within and between groups over time. When *F* values were significant, post hoc means comparisons were analysed with least significant different comparisons test.

## 5. Results

**Figure 1:**
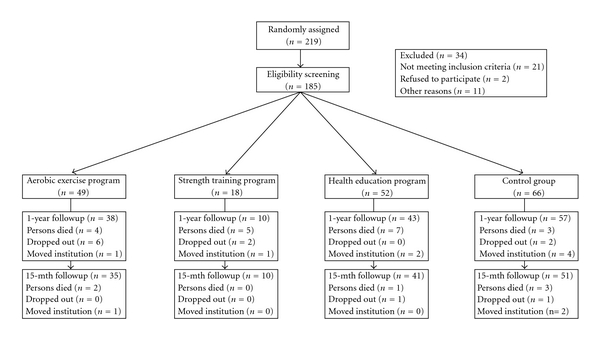
Flow diagram of enrollment, measurements.

Of the 185 participants who submitted the initial assessment, 148 (75%) participants completed pre- and postintervention and testing protocols. However, of these 148, only 137 completed the 15-month assessments (including the detraining period). During the study, 25 persons died, 11 moved out of the institutions, and 12 dropped out from the programs (Figure 1). 

Overall compliance with the exercise prescription was 78% in the AT program and 60% in the ST program. Demographics and descriptive parameters of all groups are listed in Table 1. There were no differences among groups at baseline in relevant characteristics such as age, medical condition, BMI, fitness score, HRQoL, and PA.

Table 1 also shows that after the intervention, no significant changes were observed in the control group, although the trend indicated an increase in SBP, TC, and glucose levels. However, exercise interventions groups showed a significant increase in the PA levels and improve cardiovascular variables (glucose, TC levels, and blood pressure). Similar results occured in the EH group but without statistical significance. After the detraining, we did not observe significant change in cardiovascular variables and PA, which remains significantly higher than baseline values.

Changes in functional fitness variables and body composition were presented in Table 2.

Our results showed that the biggest improvements of FFT were in the strength and flexibility tests in the ST group and in the aerobic endurance, lower body strength, and agility/dynamic balance in the AT group. The control group significantly decreases the agility/dynamic balance and the lower body strength and flexibility.

The implications of detraining in our subjects are most evident at lower-extremity body although, the upper body strength also decreased in the ST, nevertheless the values remained higher than before training. Only the agility/dynamic balance, particularly in the AT group, reverted to values lower than baseline, but not statistically significant.

## 6. Discussion

The overall results of this one-year health-promotion interventions study in institutionalized older subjects were the improvement in physical fitness, HRQoL, and cardiovascular variables, particularly in the exercise intervention groups. After the detraining, agility/dynamic balance was affected and also components from lower-extremity body, as strength and flexibility. The data also revealed a significant increase in the PA levels after intervention and declining with detraining, but remaining significantly higher than baseline values.

In accordance with the actual evidence, our results showed a major improvement in strength and flexibility in the ST group [4, 13, 33] and a significant improvement in lower body strength, aerobic endurance, and agility/dynamic balance in the AT group [34]. 

Although, previous investigations have demonstrated an age-related functional decline, these improvements are not unexpected, because, as explained earlier in the literature the specificity of the training exists even in older subjects [5, 35, 36]. Further, our results also confirm the hypothesis that one year of moderate intensity exercise interventions (twice per week) is associated with several important cardiovascular risk factors and function, namely, the declining of BP and also benefits in the HRQoL. 

Simons and Andel [34] showed that walking program is able to increase the aerobic endurance and flexibility and improve the performance of elders in the “sit-and-reach test”. In our study, the agility/dynamic balance improvements, particularly in the AT, might be related to increased lower body strength observed during the training period. According to Marzilli et al. [33] better balance was due to rises in lower body strength in older adults. Additionally, specific balance activities used in the present study may possibly have stimulated additional benefits. It was not possible to establish the independent effect of exercise programs on flexibility, because all of the programs also included flexibility exercises [37]. 

In this sense, Peterson et al. [7] state that the AT, as well as being used in the elderly, has excellent health benefits, increasing cardiofitness and strength and reduces the risk factors associated with CVD and metabolic diseases [38]. However to promote and maintain physical health and functional independence is also necessary to preserve and increase muscle strength through ST [39, 40]. ST compensates the loss of muscle and bone mass associated with aging, reducing the risk of fractures, and otherwise maintains the functionality and flexibility postural stability, thus reducing the risk of falling [41, 42]. 

According to our results, we observed that ST was the most effective way to improve plasma lipoprotein lipid profiles and also improve insulin action, which results in one significant decrease of glucose, triglycerides, HDL-cholesterol and TC. We observed, as Durstine et al. [43] suggested, that exercise training lasting longer than 12 weeks is more likely to increase plasma HDL cholesterol and triglycerides in a dose-dependent manner. 

In this way, PA is a most appropriate intervention in a collaborative approach to the management of abnormal blood lipids and should be undertaken at moderate to high intensity, 5 to 7 days/week for at least 30 min/day [44, 45]. Furthermore, to achieve changes in lipids or weight, the majority of the studies indicate that changing dietary behaviour as well as performing vigorous exercises is fundamental and yields the best effects [10, 46]. 

Although the mechanisms underlying the lowering effect of exercise on BP are not fully understood, the recent ACSM and AHA [47] guidelines recommend light- to moderate-intensity lifestyle physical activities to optimize health, and moderate or high-intensity exercise may be required to produce adaptations in the cardiovascular system and in CVD risk factors. These changes are of importance since hypertension, a common disease of older individuals, is associated with an increased incidence of all-cause and CVD mortality and morbidity such as, stroke, coronary heart disease, and renal failure [48].

Barger and Muldoon [49] concluded that hypertensive labeling was associated with poorer HRQoL as measured by global self-rated health. Kuo et al. [50] in a longitudinal study concluded that hypertension and diabetes were associated with a significantly faster pace of decline on the perceived HRQoL and elders with diabetes had a faster pace of decline in activities daily living functioning than nondiabetic subjects.

Many studies have examined the effects of exercise on several cardiovascular risk factors, depending on the type, intensity, duration of exercise, and participants' age and functional status and different methodologies used to evaluate older population, and results have been discordant. In fact these positive cardiovascular health changes are usually more associated with reductions of body weight and dietary fat reductions [46, 51]. Although our subjects were instructed to maintain their normal dietary routines throughout the protocol period, this was not strictly controlled. It is possible that the participants in our study increased their energy intake in response to the energy demands of exercise training. 

The data available in the literature generally support the conclusion that older adults improve their plasma lipoprotein lipid profiles with PA, increase in plasma HDL cholesterol levels, and have diminutions in triglyceride and cholesterol levels [52, 53]. Nevertheless, these changes may be secondary to training-induced reductions in body fat scores [54, 55]. 

Nevertheless, we should highlight that our participants experienced significant changes in lipid levels. Pescatello et al. [56] showed that the existence of greater amounts of PA and of low intensity is associated with a more favorable lipid profile in the elderly. We also observed that higher BMI is also associated with reduced PA and functional capacity, suggesting that the risk of obesity may be explained partially by the adverse effects of inactivity. While weight loss has been shown to decrease multiple cardiovascular risk factors [57], the direct benefit of weight reduction on CVD risk remains unproven and unclear. Our data adds evidence to the role of PA as prevention to the CVD risk factor in elders, which is in accordance with the expected effects of health promotion intervention with PA [58]. 

Overall, the studies related to the risk of CVD and PA indicate that the relationship is causal and most likely to provide evidence of a dose-response relationship, showing that there is a decrease in CVD mortality in subjects with a more active lifestyle [59]. These results have been demonstrated in a variety of populations and using a diversity of methods of assessment of PA. This variability of measuring makes it difficult to compare results between studies. This sustains the need for more studies with different protocols and considering these different variables to clarify these relationships.

Our results clearly clarified the potential of an appropriate regimen of regular PA among elders, particularly with the analysis of the changes in the detraining period. Regarding the effects of detraining, like in previous studies, our results demonstrated that strength [11, 60] and flexibility [13, 22, 61] gains after training decreased after 3 months of detraining, nevertheless the values remained significantly higher than before training. However, agility/dynamic balance in the AT group reverted to lower values than the baseline. In opposition, we observed that aerobic endurance was the less affected component by detraining period [13, 22].

Training cessation among elders is generally associated with the loss of functional ability. Some studies indicate that the morphological and functional adaptations to training may disappear, even after short periods of detraining [11, 62]. Therefore, like our data support, long-term exercise programs undertaken prior to activity cessation may offer functional protection [14, 63]. Moreover, moderate to higher intensity training may maintain the gains for longer periods after training ends.

On the other side, it is difficult to explain the discrepancy between the upper and lower body response to short-term detraining in this small sample. One possible explanation is that the initial training response was greater for lower-extremity strength than for upper-extremity strength and our subjects may have been less physically active than in some studies. Moreover, when we analyzed the results of 1-year followup, it was found that elders in the control group decreased their levels of all parameters of the FFT, except for upper strength and flexibility, possibly because the muscles of the upper extremity are the most used and this could result in better physical function [42, 64]. This difference may also be explained by the activities performed in the leisure time, morphological differences in the connective and skeletal tissues, and hormonal differences [65]. 

According to Misic et al. [66] muscle function is the most important predictor physical function of the lower extremities in the elderly. Furthermore, aerobic endurance and fat mass are secondary predictors, which are widely associated with the functional capacity to perform various activities of daily living [6].

These underlying results show that the negative effects of detraining reduce the functional capacity of the elderly and hence reflect on their HRQoL, highlighting that PA should be practiced regularly. Supporting these results, the literature shows that the elderly who became active had a better HRQoL than their peers who remained sedentary Wu et al. [67], which indicates the great value of exercise interventions in the elderly.

Strengths of this study include the objective measure of PA, through 15 months of study with a 1-year intervention, controlled experimental design, and most importantly the opportunity to compare different intervention strategies. However, this study has some important limitations: first, there was no assessment of subjects' dietary regimens throughout the investigation; second, PA was not assessed during detraining and PA patterns could change during that time, due to seasonality [68]; third, there was an imbalance in group sizes. Finally, probably the main limitation of this study is that participants were selected from long-term care institutions and they may not represent all older adults, in particular those who are physically independent and community-dwelling elders. Further research is needed to determine whether diet and cardiovascular risk modification ameliorate cognitive and functional decline in elderly people.

In conclusion, results highlight that a period of 3 months of detraining following 1-year of health intervention programs significantly impairs the major part of the favorable functional changes obtained after training. Agility/dynamic balance, lower body strength, and flexibility were the components of functional fitness most affected by detraining in the AT. Therefore, our data reinforce the idea that detraining affects physical fitness and elders should be engaged in a systematic exercise program throughout life in order to maintain or improve functional performance [69, 70].

Thus, PA and(or) exercise prescriptions should emphasize components related to the maintenance of functional capacity and independence; these will also improve cardiovascular health.

Since low PA levels are health risk factors for ageing subjects [71], health professionals need to be more effective promoting PA as an integral component of a healthy lifestyle. Elders who undertake PA have better chances of maintaining or increasing physical function. Besides they can also improve their social life opportunities.

## Figures and Tables

**Table 1:** Baseline, postintervention, and postdetraining values for cardiovascular variables and delta changes.

Age (years)		CG (*n* = 51)		AT (*n* = 35)		ST (*n* = 10)		HE (*n* = 41)	
		77.6 ± 6.9	% Change	81.0 ± 7.1	% Change	75.1 ± 8.3	% Change	81.6 ± 6.7	% Change
PA (counts/h)	B-Δ_1_	11591 ± 7739	−5.8	10953 ± 6219	**62.4**	11938 ± 7920	**43.1**	12091 ± 8519	**15.3**
	AI-Δ_2_	10915 ± 7112		17761 ± 8218^∗a^	−13.1	17053 ± 5519^∗a^	−1.7	13933 ± 6902	−1.8
	AD-Δ_3_			15461 ± 8008	**41.5**	16756 ± 8765	**40**	13676 ± 7861	**13.1**

SBP (mmHg)	B-Δ_1_	138.8 ± 11	−0.7	133.4 ± 22	−1.3	130.3 ± 15	−3.7	128.5 ± 13	−1.5
	AI-Δ_2_	139.3 ± 12		131.6 ± 17	−2.5	125.1 ± 6	2.8	126.5 ± 13	−2.3
	AD-Δ_3_			128.3 ± 17	−3.8	128.6 ± 16	−1.4	123.5 ± 14	−3.9

DBP (mmHg)	B-Δ_1_	80.7 ± 9.4	−0.8	82 ± 10	−2.4	80.5 ± 11	−2.1	76.9 ± 10.2	−1.3
	AI-Δ_2_	80.0 ± 9.6		80 ± 4 ± 9	0.8	78.8 ± 15	−0.4	75.9 ± 11	3.7
	AD-Δ_3_			80.7 ± 11	−1.5	78.4 ± 8	−2.6	78.7 ± 10	2.4

Cholesterol	B-Δ_1_	181 ± 32	3.4	191 ± 28^a^	−**14.1**	182 ± 20^a^	−**24**	178 ± 18	−2.2
	AI-Δ_2_	187.3 ± 36		164 ± 37	4.2	138 ± 23^b^	5.1	174 ± 18	−1.7
	AD-Δ_3_			171 ± 24	−**10.2**	145 ± 31	−**20.2**	171 ± 13	−3.9

HDL (mg/dL)	B-Δ_1_	51.2 ± 9.6	−2.5	48.9 ± 7.1	4.2	53.6 ± 12	−0.7	55.6 ± 12	−1.9
	AI-Δ_2_	49.9 ± 9.8		50.9 ± 11	−5.5	53.2 ± 6	−5.8	54.5 ± 10	0.0
	AD-Δ_3_			48.1 ± 8	−1.6	50.1 ± 5	−6.5	54.5 ± 10	−1.9

Glucose (mg/dL)	B-Δ_1_	108 ± 30	0.9	106 ± 28	−2.7	115 ± 39	−**11.2**	107 ± 31	0.1
	AI-Δ_2_	109 ± 24		103.1 ± 22	0.6	102 ± 17	0.8	107 ± 29	−6.5
	AD-Δ_3_			103.8 ± 32	−2.1	103 ± 12	−**10.4**	100 ± 15	−6.6

HRQoL	B-Δ_1_	62.0 ± 13.4	−6.6	64.9 ± 14.2	**8.2**	60.1 ± 15.1	**13.8**	58.3 ± 12.6	5.2
	AI-Δ_2_	57.9 ± 9.8		70.2 ± 6.7	0.8	68.4 ± 11.1	−3.1	61.3 ± 9.4	−3.5
	AD-Δ_3_			69.5 ± 7.5	7.1	66.3 ± 9.4	**10.1**	59.2 ± 10.4	1.7

CG: control group; AT: aerobic training; ST: strength training; HE: health education.

Univariate analysis of variance.

^a^Significant difference versus CG, *P* < .05.

^b^Significant difference versus HE, *P* < .05.

*Significant difference versus baseline, *P* < .05.

^#^Significant difference versus after intervention, *P* < .05.

B: baseline; AI: after intervention; AD: after detraining

Mixed-model ANOVA with repeated measures.

^$^Significant, *P* < .05.

**Table 2:** Baseline, postintervention, and postdetraining values of functional fitness test and delta changes.

		CG (*n* = 51)	% Change	AT (*n* = 35)	% Change	ST (*n* = 10)	% Change	HE (*n* = 41)	% Change
Lower body strength (no. in 30 s)	B-Δ_1_	14.8 ± 4.5	**−12.5**	14.6 ± 4.7	**27.1**	12.8 ± 5.9	**25.8**	13.8 ± 5.4	7.3
	AI-Δ_2_	13.3 ± 3.7		18.5 ± 5.5^∗a^	**−11.4**	16.1±5.5*	−7.4	14.9 ± 4.4	5.3
	AD-Δ_3_			16.6 ± 5.2^#^	**14.2**	14.9 ± 5.3^#^	**16.4**	15.7 ± 5.2	**13.7**

Upper body strength (no. in 30 s)	B-Δ_1_	18.2 ± 6.7	1.1	20.7 ± 9.9	3.4	14.5 ± 6.5	**49.6**	17.3 ± 9.8	9.3
	AI-Δ_2_	18.4 ± 5.9		21.3 ± 9.1	−1.3	21.7 ± 7.2*	−9.2	18.9 ± 8.3	−7.4
	AD-Δ_3_			21.1 ± 8.9	2.1	19.7 ± 6.9^#^	**35.8**	17.5 ± 8.6	1.2

Aerobic endurance (no. in 2 min)	B-Δ_1_	99.2 ± 21.2	**−7.4**	102.3 ± 21.9	**9.3**	107.2 ± 13.5	4.4	96.7 ± 19.7	3.7
	AI-Δ_2_	92 ± 23.1*		111.8 ± 17^∗a^	−7.3	111.9 ± 11.5^a^	0.4	100.3 ± 18.3	6.1
	AD-Δ_3_			104.1 ± 18.7	2.2	112.3 ± 12.5	4.8	106.4 ± 18.9	**10.1**

Lower body flexibility (in. from toe)	B-Δ_1_	−1.2 ± 4.9	**−125.2**	−1.4 ± 5.7	**21.4**	−2.4 ± 4.1	**45.8**	−2.0 ± 4.9	5.1
	AI-Δ_2_	−2.7 ± 4.4*		−1.1 ± 4.8^a^	**−27.3**	−1.2 ± 3.9*	**−25**	−1.9 ± 3.8	6.7
	AD-Δ_3_			−1.4 ± 5.6^#^	0.1	−1.5 ± 4.0^#^	**37.5**	−1.4 ± 4.2	0.3

Upper body flexibility (from fingers)	B-Δ_1_	−9.6 ± 8.2	−1.4	−10.8 ± 8.9	**15.7**	−15.4 ± 12.2	**22.7**	−10 ± 8.1	1.1
	AI-Δ_2_	−9.5 ± 6.6		−9.1 ± 6.5*	−8.7	−11.9 ± 8.7*	−0.8	−9.9 ± 7.1	−3.2
	AD-Δ_3_			−9.9 ± 6.9	8.8	−12 ± 9.9	**22.1**	−10.2 ± 7.8	−2.1

Agility/dynamic balance (sec.)	B-Δ_1_	13.4 ± 7.4	**11.4**	12.7 ± 7.7	**−22.8**	12.6 ± 5.8	−9.5	13.2 ± 6.9	3.8
	AI-Δ_2_	14.9 ± 7.3		9.8 ± 5.7^∗ab^	**41.8**	11.4 ± 4.7^d^	9.6	13.7 ± 5.2	0.7
	AD-Δ_3_			13.9 ± 6.2^#^	9.4	12.5 ± 4.9	−0.8	13.8 ± 5.8	4.5

BMI (Kg/m^2^)	B-Δ_1_	26.9 ± 3.1	**11.6**	25.7 ± 3.1	**−8.9**	24.6 ± 0.2	1.2	25.1 ± 2.9	**13.9**
	AI-Δ_2_	29.8 ± 3.4^b^		23.4 ± 3.0^ab^	−2.1	24.9 ± 3.1	0.8	28.6 ± 2.7	−2.8
	AD-Δ_3_			22.9 ± 4.1^ab^	**−10.9**	25.1 ± 4.3	2.1	27.8 ± 3.2	**10.6**

Body fat mass (%)	B-Δ_1_	22.1 ± 5.8	6.8	21.9 ± 6.4	**−9.6**	22.5 ± 8.8	2.6	17.2 ± 6.2	**16.8**
	AI-Δ_2_	23.6 ± 4.1		19.8 ± 5.6	1.5	23.1 ± 7.2	1.7	20.1 ± 7.6	0.5
	AD-Δ_3_			20.1 ± 6.8	−8.1	23.5 ± 8.6	4.4	20.2 ± 8.4	**17.4**

CG: control group; AT: aerobic training; ST: strength training; HE: health education.

Univariate analysis of variance.

^a^Significant difference versus CG, *P* < .05.

^b^Significant difference versus HE, *P* < .05.

*Significant difference versus baseline, *P* < .05.

^#^Significant difference versus after intervention, *P* < .05.

B: baseline; AI: after intervention; AD: after detraining.

Mixed-model ANOVA with repeated measures.

^$^Significant, *P* < .05.
